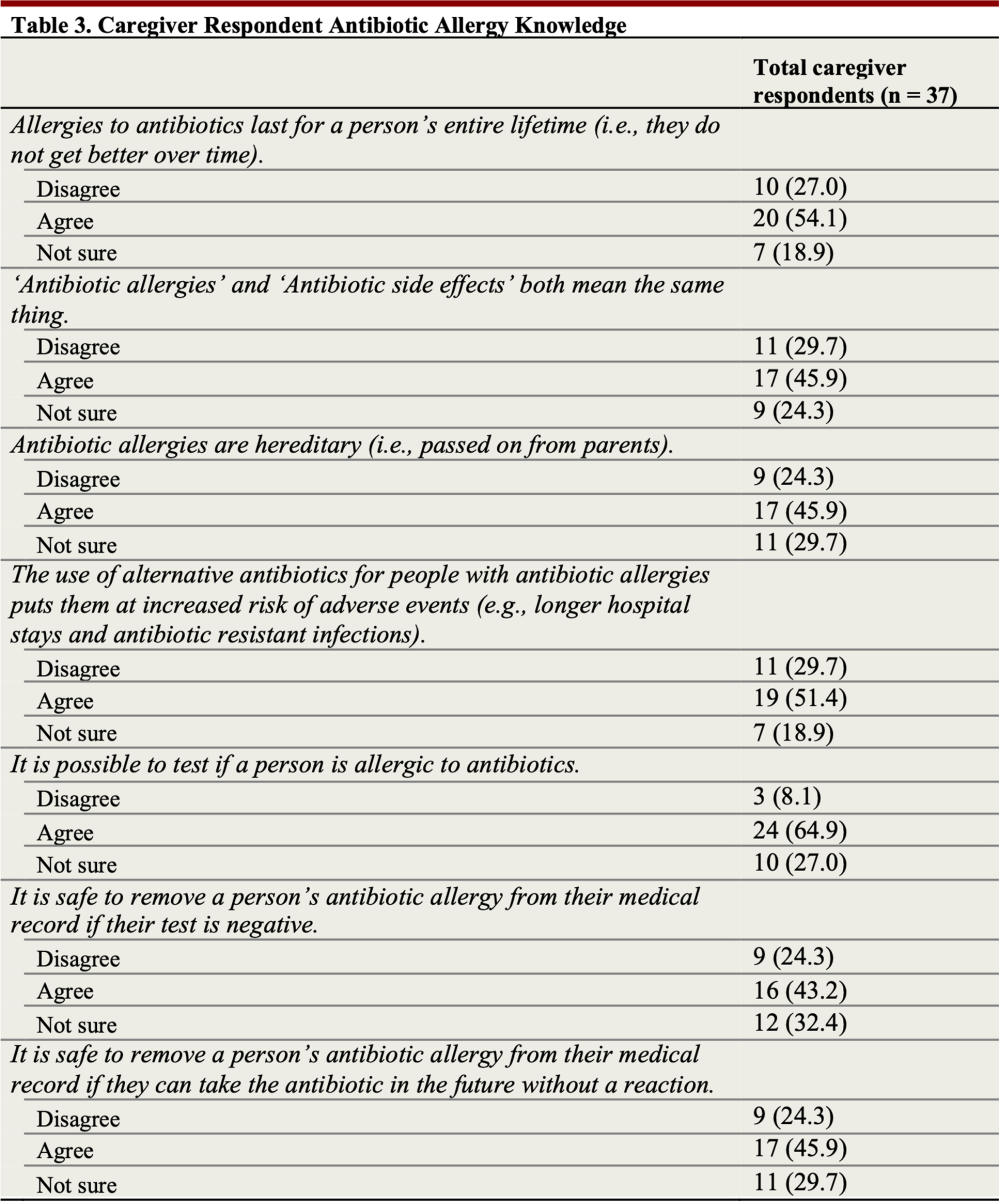# 343 A Predictive Modeling Approach to Reduce Pediatric Central Line-Associated Bloodstream Infections

**DOI:** 10.1017/ash.2026.10683

**Published:** 2026-06-23

**Authors:** Alistair Thorpe, Rachael Lee, William Petty, Angela Fagerlin, Valerie Vaughn, Julie Szymczak

**Affiliations:** 1 University of Utah; 2 University of Alabama at Birmingham; 3 University of Utah School of Medicine

## Abstract

**Background:** Approximately one in ten US children report an allergy to antibiotics. However, ~95% of these pediatric allergy labels are inaccurate upon evaluation. Inaccurate allergy labels lead to suboptimal antibiotic prescribing, increasing patients’ risk of treatment failure and adverse effects. Successfully removing inaccurate pediatric allergy labels (i.e., delabeling) requires engagement from primary caregivers. This study examined caregiver (e.g., parents, stepparents) perceptions and attitudes toward delabeling in pediatric care. **Methods:** We invited US adults to an online survey about antibiotics (March-April, 2024). Respondents identifying as a primary caregiver for a child with an antibiotic allergy answered questions about the child’s allergy (e.g., type, severity), their willingness to try methods to assess whether the child was non-allergic (oral medication vs. skin-scratch challenge), and their anticipated reaction to a negative skin-scratch test result for the child (i.e., indicating no allergy). **Results:** Table 1 shows characteristics of the 37 caregivers of a child with an antibiotic allergy, 31 described the child’s allergies as mild/moderate (83.8%), 5 as severe (13.5%), and 1 as life-threatening (2.7%). Penicillin was the most common allergy (27 of 37 [73.0%]). More caregivers were willing to try skin-scratch testing (33 of 37 [89.2%]) than were willing to try oral challenge (26 of 37 [70.3%], p=.02). Yet, 21.6% of caregivers (n=8) would not accept the results of a negative skin-scratch test for the child and 29.7% (n=11) would not agree to remove the allergy from the child’s medical record based on that test. Almost all caregivers (35 of 37 [94.6%]) said they would try to re-add the child’s allergy onto their medical record if removed following a negative skin-scratch test. Caregivers listed various concerns about delabeling in free-text boxes including fear of harm or distress to the child, negative past experiences, distrust of clinicians, and belief that, as caregivers, they know the child best and should not knowingly do something that could harm them. Other concerns included keeping allergy labels as a safety net, worrying allergies could return, and preferring to be ‘safe than sorry’. **Conclusions:** In this survey of US adults, caregivers of children with self-reported antibiotic allergies expressed hesitancy toward delabeling efforts. Findings suggest increased testing efforts alone are likely insufficient as caregiver attitudes may present a major barrier to effective antibiotic allergy delabeling in pediatric care. Strategies for addressing caregiver concerns that persist after negative test results are needed to ensure safe, guideline-concordant antibiotic prescribing through childhood and beyond.

**Figure:**
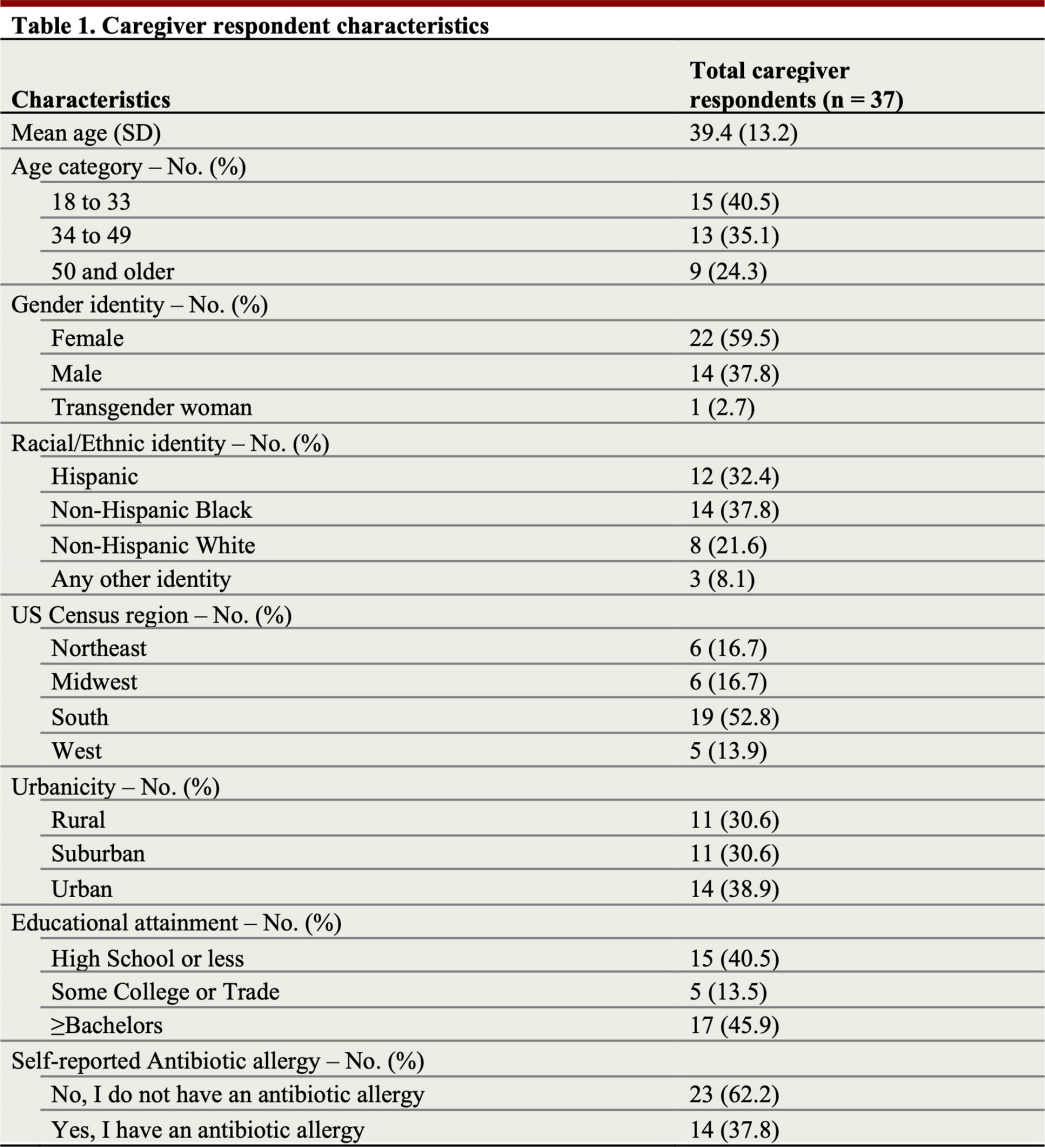

**Figure:**
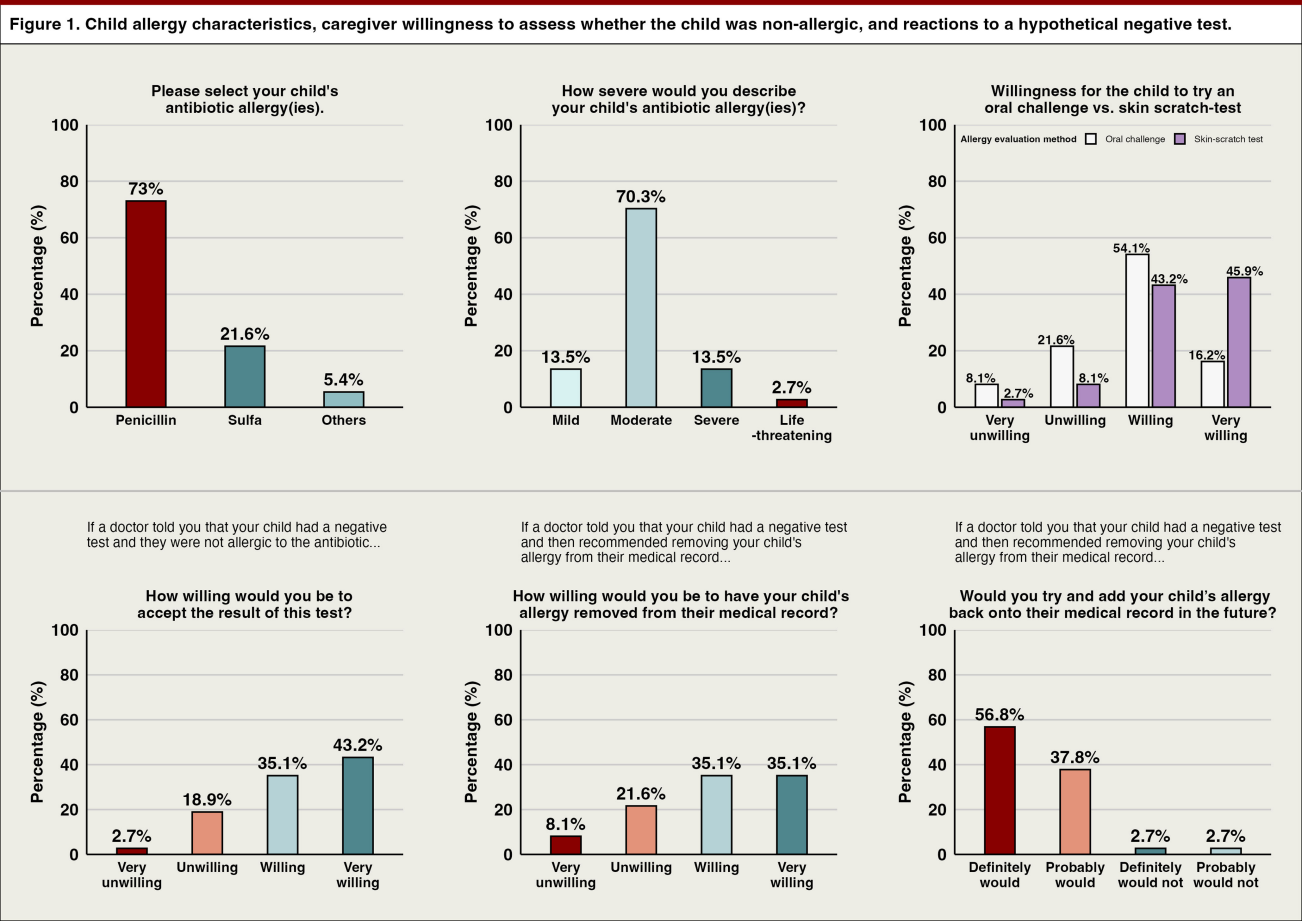

**Figure:**
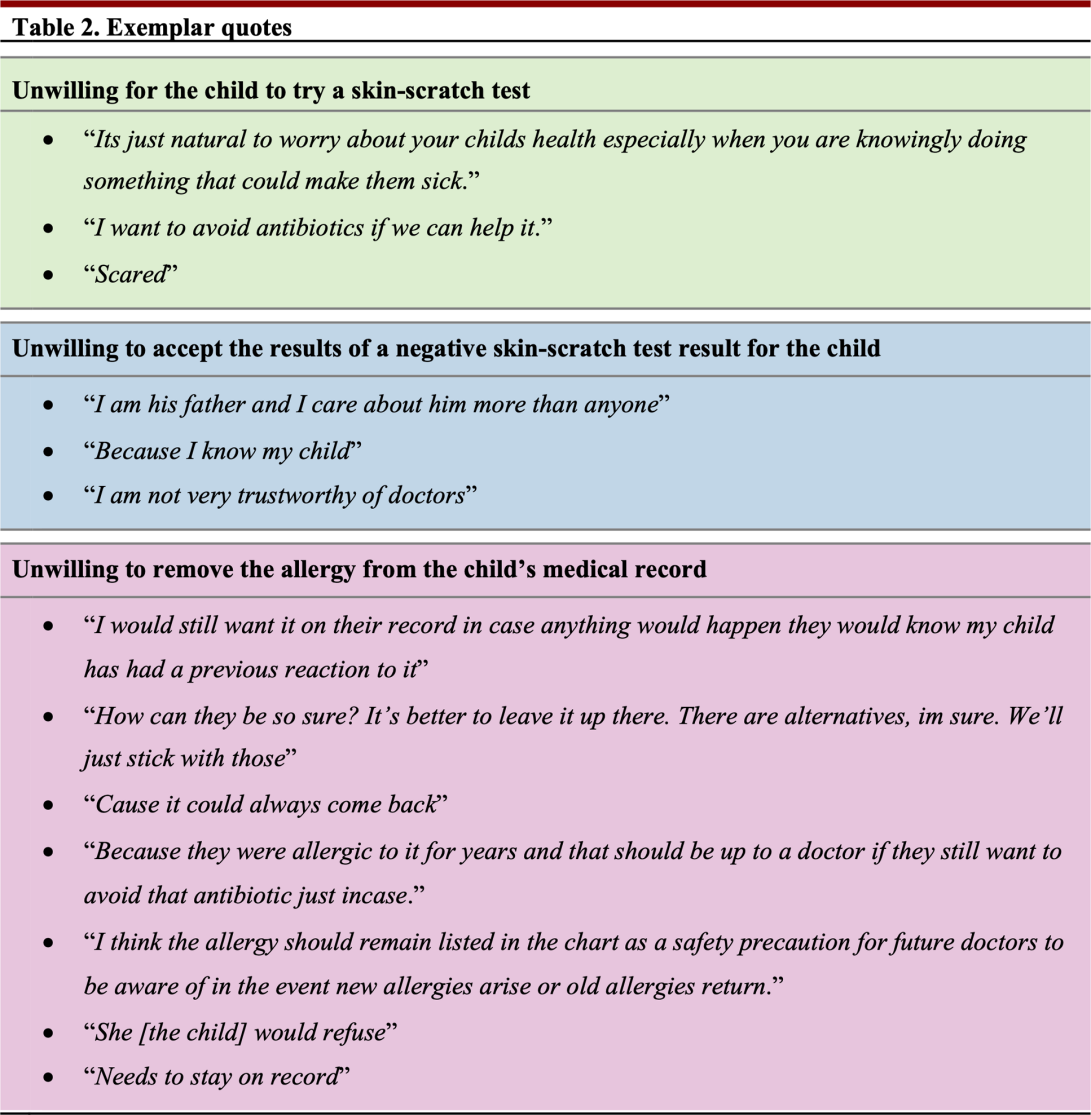

**Figure:**